# Newly listed firms’ M&A activities data and their VC-backing data

**DOI:** 10.1016/j.dib.2016.11.015

**Published:** 2016-11-09

**Authors:** Ting Cao

**Affiliations:** School of Management, Xi’an Jiaotong University, China

## Abstract

This article contains hand collected and matched data on the VC investment situation in newly listed firms in SME and ChiNext board from 2009 to 2012 and the corresponding M&A activities data undertaken by these newly listed firms as acquirers in three years following initial public offering. Mentioned data are related to the research article “Heterogeneous Venture Capital, M&A Activity, and Market Response” (W. Li, T. Cao, Z. Feng, 2016) [1].

**Specifications Table**

**Table:** 

Subject area	*Corporate finance and governance*
More specific subject area	*Venture capital investment time, newly listed firms’ M&As occurrence and frequencies*
Type of data	*Table, Excel file*
How data was acquired	*CSMAR database*
	*PEDATA database*
	*WIND database*
	*Prospectus of newly listed firms*
Data format	*Filtered*
Experimental factors	*Financial firms (Industry code J) are eliminated from the samples, because the regulations are different in the financial industry.*
	*The M&A sample is selected using following criteria: the acquirer company undergoes an IPO from 2009 to 2012 on the ChiNext or SME board; M&A types include asset acquisition and merger. The acquisition subject is only limited to stock when it refers to an asset acquisition.*
	*Observations without complete financial information are deleted.*
Experimental features	*Obtain information from public database and hand-collected data.*
Data source location	*China*
Data accessibility	*Data is within this article*

**Value of the data**

• Hand collected data from public firms’ IPO prospectus supplements the missing data in existing PEDATA database as for VC׳s investment.
• Incorporate the firm׳s VC investment data with their M&A activities data.
• The data can be useful for other researchers investigating VC׳s influence on firm׳s M&A activities.

## 1 Data

The panel data consist of 781 listed firms ranging from the first year following IPO to the third year following IPO totaling 2267 observations. Definition of variables involved in the data and the basic statistic description of the data are shown in Table 1, Table 2 respectively.

## 2 Experimental design, materials and methods

We first obtain VC’ investment in newly public firms listed on ChiNext board or SME board from 2009 to 2012 from PEDATA database, including VC׳s total investment amount, first entry time, shareholding at IPO data.Given the VC institution criteria may vary across different database, to make our VC institution list more convincing, we cross check the VC investment data using WIND database to make sure every VC institution we include in our research sample appears in both databases [1].As for the missing data for each VC observation, we hand collected them from the listed firm׳s IPO prospectus.After the VC investment data was ready, we filtered M&A activities data for each newly public firms listed on ChiNext board or SME board from 2009 to 2012 from CSMAR database. Then we matched it with prepared each sample firm׳s VC investment data.

## Figures and Tables

**Table 1:** Definition of variables.

Variable name	Data type	Definition
MA	Binary variable	MA is short for merger and acquisition. It measures whether firm i undertakes M&A as an acquirer in year t. Dummy=1 if firm i in year t undertakes M&A as an acquirer, otherwise 0.
MAnumber	Count variable	MAnumber measures the number of M&A activities undertaken by firm i in year t.
VC	Binary variable	VC is short for venture capital. Dummy =1 if VC holds firm i׳s share at the time of IPO.
VCshare	Continuous variable	VC׳s pre-IPO investment share.
Period	Weighted average continuous variable	Weighted average of VCs’ investment periods before IPO backing firm i, measured in month.

**Table 2:** Statistical description of variables.

Variable	Number of observations	Mean	Min	Max	Std.
MA	2267	0.2338	0.0000	1.0000	0.4233
MAnumber	2267	0.3167	0.0000	8.0000	0.6828
VC	2267	0.4936	0.0000	1.0000	0.5001
VCshare	2267	6.6309	0.0000	50.0000	9.2453
Period	2267	14.7660	0.0000	117.0000	19.4613
